# Flow injection analysis of iron in rain water with thiocyanate and surfactant

**DOI:** 10.1155/S1463924697000072

**Published:** 1997

**Authors:** A. N. Tripathi, S. ehikhalikar, K. S. Patel

**Affiliations:** School of Studies in Chemistry Pt. Ravishankar Shukla University Raipur M. P. 492 010 India

## Abstract

This paper explains a new procedure for flow injection analysis (FIA) determination of iron in rain water based on the colour reaction of Fe^3+^ with thiocyanate ions in the presence of the cationic surfactant cetylpyridinium chloride (CPC). The value of apparent molar absorptivity of the complex in terms of iron is (2.00) x 10^4^ l mole^-1^ cm^-1^ at an absorption maximum of 490 nm. The detection limit of the method is 8 ppb Fe. The sample throughput is 90 samples/h at a flow rate of 4.0 ml/min. The reaction mechanism, optimization of FIA variables, and effect of various types of surfactant are described. None of the tested anions and cations interfered with the determination of iron. The method was used for the quantification and flux determination of iron in rain water.


Journal of Automatic Chemistry, Vol. 19, No. 2 (March–April 1997) pp. 45–50

### Flow injection analysis of iron in rain water with thiocyanate and surfactant

**A. N. Tripathi, S. Chikhalikar and K. S. Patel***

*School of Studies in Chemistry, Pt. Ravishankar Shukla University, Raipur-492 010, M. P., India*

*This paper explains a new procedure for flow injection analysis (FIA) determination of iron in rain water based on the colour reaction of* $Fe^{3+}$ *with thiocyanate ions in the presence of the cationic surfactant cetylpyridinium chloride (CPC). The value of apparent molar absorptivity of the complex in terms of iron is* $(2{\cdot}00) \times 10^4$ *l mole*$^{-1}$ *cm*$^{-1}$ *at an absorption maximum of* 490 *nm. The detection limit of the method is* 8 *ppb Fe. The sample throughput is* 90 *samples/h at a flow rate of* 4·0 *ml/min. The reaction mechanism, optimization of FIA variables, and effect of various types of surfactant are described. None of the tested anions and cations interfered with the determination of iron. The method was used for the quantification and flux determination of iron in rain water.*

### Introduction

Iron is emitted to the troposphere from both natural and anthropogenic sources [1–2]. The oxidation state of iron is an indicator of tropospheric redox potential and participants in a variety of reactions, for example oxidation of inorganic and organic compounds, catalytic oxidation of S(IV) to S(VI) in the rain droplet phase, carrier for the trace elements [3–5]. Sophisticated techniques, such as graphite furnace AAS, ICP-MS, anodic striping voltammetry are generally used for monitoring iron in atmospheric samples at ppb levels, but these involve drawbacks, for example, tedious procedure, non-linearity of the calibration curve and matrix interferences [6–10]. Organic reagents, i.e. 1, 10-phenanthroline, ferrozine, (3(2-pyridil)-5,6-bis(4-phenyl-sulfonic acid)-1,2,4-triazine, disodium salt), 2-(5-nitro-2-pyridylazo)-5-(N-propyl-N-sulfopropylamino)phenol, have been used for flow injection analysis (FIA) determination of iron [11–14]; these have drawbacks in terms of a narrow pH range and interference from some ions. Other reported procedures for the FIA determination of iron required prior preconcentration (15–17). This paper describes an attempt to develop a simple, rapid FIA procedure for the spectrophotometric determination of iron in a large number of rain water samples by reacting $Fe^{3+}$ with thiocyanate ions in the presence of cationic surfactant (cetylpyridinium chloride [CPC]). The procedure overcomes most of drawbacks found in routine FIA methods. In addition, its sensitivity is comparable to the sophisticated technique employed for determination of iron.

* Correspondence to K. S. Patel.

### Experimental

### *Reagents*

All chemicals and reagents used were of AR grade (E. Merck). The working solution of iron was prepared by diluting the standard solution containing 1·000 g Fe(III)/l with deionized double distilled water to an appropriate dilution. All solutions used were degassed and filtered. Seven per cent w/v (1·3 M) fresh ammonium thiocynate, 0·07%, w/v ($1{\cdot}95 \times 10^{-3}$ M) cetylpyridinium chloride and 0·05 M HCl+0·05 M $H_2SO_4$ (1 : 1) solutions were used and aspirated through silicon tubes C (1·14 mm), $R_1$ (0·51 mm), $R_2$ (0·51 mm) and $R_3$ (0·38 mm), respectively, at flow rate of 4·0 ml/min.

### *Apparatus*

A Tecator FIA analyser type-5012 with two peristaltic pumps of constant speed (48 cycles/min), equipped with ALPKEM UV-VIS spectrophotometer type-510 (matched with 5·5 mm flow cell), was used.

### *Procedure*

*Collection of water samples:* Samples were collected in clean plastic buckets as prescribed by GAW, Switzerland. The buckets were installed on the roof of a house about 5 m from ground level. Collections were made at 13 sites (extending over $300 \times 300$ km$^2$ in the east Madhya Pradesh, central India). The level of rain water was measured, filtered, and transferred into a 1 l clean plastic bottle. The details of meteorological parameters were traced in the bottle and dispatched to the laboratory for analysis.

*Determination of ferric iron:* The carrier (deionized double distilled water) and reagent solutions (ammonium thiocyanate, CPC, and acid) were run into chemifold with peristaltic pumps of constant speed (48 cycle/min). Optimum value of FIA variables, i.e. rise, injection, and delay time were set in the analyser. A base line of zero absorbance (at wavelength 490 nm) of the flowing stream was recorded. A 370 µl aliquot of the filtered standard solution containing 30–800 ppb ferric iron was injected into a flowing stream with a flow rate of 4·0 ml/min. The signal peak height of the analyte was recorded at absorption maximum, 490 nm. Similarly, the filtered rain water samples were injected and the concentration of iron in the rain water was determined using a linear least squares equation:

$$100\,Y = 1{\cdot}90\,X - 8{\cdot}90$$

$Y$, and $X$ denote the peak height of signal, and concentration of Fe(III) in ppb, respectively.

0142-0453/97 $12·00 © 1997 Taylor & Francis Ltd

*Determination of the total iron (ferrous and ferric iron)*: A 5 ml filtered rain water sample was taken in a plastic test tube and oxidized with 0·2 ml concentrated nitric acid. The sample solution was injected by recording the signal peak height as above. Similarly, the signal peak height of the reagent blank was also recorded (as the nitric acid contains some iron) and the net iron content of the rain water was computed.

### Results and discussion

*Colour reaction*

Iron(III) spontaneously reacts with thiocyanate ions to give a variety of red-orange coloured complexes in acidic solutions:

$$Fe^{3+} + n\, SCN^- \rightleftharpoons [Fe(SCN)_n]^{3-n}$$

$n$ denotes the number of thiocyanate ions. The value of $n$ can vary from 2 to 6 depending upon the chemical environment. In the absence of surfactant, the molar ratio of $Fe^{3+}$ to $SCN^-$ in the complex was found to be 1 : 2. However, in the presence of cationic surfactant, i.e. cetylpyridinium chloride (CPC) or cetyltrimethylammonium bromide(CTAB), formation of a higher thiocyanato complex with a deeper colour ( $\geqslant$ 5 fold) was observed (see figure 1).

$$Fe^{3+} + n\, SCN^- + m\, CP^+ \rightleftharpoons \{[Fe(SCN)_n](CP)_m\}^{(3+m)-n}$$

The values of $n$ and $m$ were determined using the curve-fitting method by plotting $\log(h_{eq}/h_{max} - h_{eq})$ versus log molar concentration of the reagent injected. The values were 6 and 2, respectively. The existence of the ion associated complex $(CP)_2[Fe(SCN)_6]^-$ is expected in the flowing stream. The surfactant was assumed to act as a counter ion and to enhance the formation constant of the complex with increased sensitivity.

*Effect of FIA variables*

The effect of FIA variables, i.e. tube diameter, coil length, rise time, injection time, and delay time, on the determination of $Fe^{3+}$ were optimized. At least 1·0, 15·0, and 25·0 s of rise, injection, and delay time, respectively were needed to get maximum signal peak height. Further increases in these did not produce an adverse effect. The sample throughput was 90 samples/h at a flow rate of 4·0 ml/min. The optimum diameter of silicon tubes C, $R_1$, $R_2$, and $R_3$ were found to be 1·14, 0·51, 0·51, and 0·38 mm, respectively. The optimum coil length of the merging zone was 30 cm (diameter = 0·5 mm).

*Effect of acids, thiocyanate, and surfactants*

The effects of acids (HCl, $H_2SO_4$, $HNO_3$) on the determination of ferric iron were tested. All were found to be adequate, but with HCl and $HNO_3$ a prolonged return time was needed to get the smoth base line. For simplicity an equimolar mixture of HCl and $H_2SO_4$ was used and the optimal acidity range was found to be very wide over acidity 0·005–5·0 M HCl + $H_2SO_4$. Similarly, the acidity of the sample solution injected over pH values 0·7–9·0 did not affect the signal peak height when the

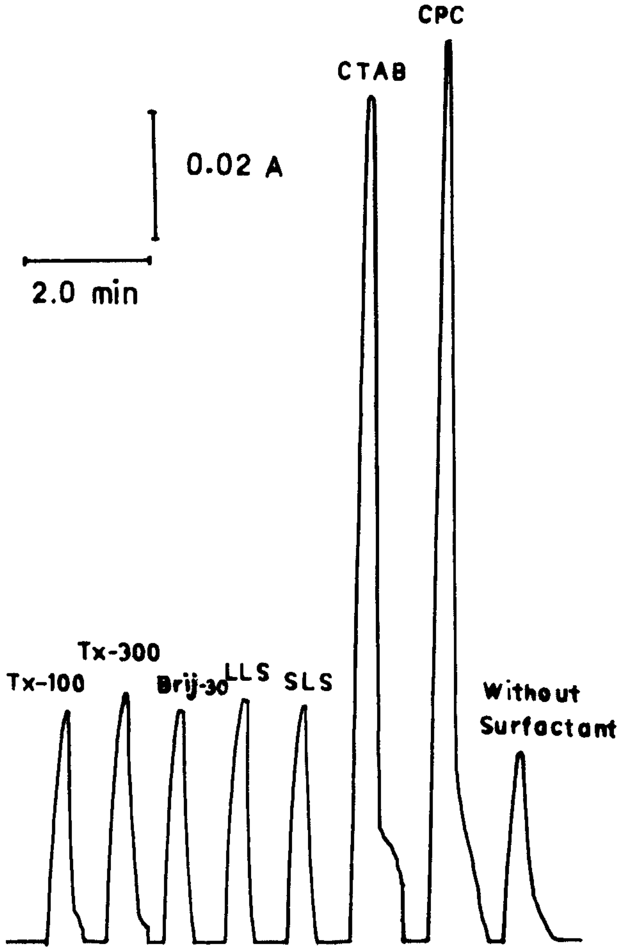

*Figure 1. Signal peak height recorded for standard solution of Fe(III) (700 ppb) with different surfactants. LLS = Lithium lauryl sulphate; SLS = Sodium lauryl sulphate; CTAB = Cetyltrimethylammonium bromide; CPC = Cetylpyridinium chloride; TX-100 = Triton X-100; TX-300 = Triton X-300; Birj 30 = polyoxyethylene(4) lauryl ether.*

mixed acid (containing 0·05 M HCl + 0·05 M $H_2SO_4$) was run through the silicon tube ($R_2$). At least 0·8 M of $NH_4SCN$ solution was needed to get maximum peak height and its further addition up to 2·6 M had no adverse effect. The effects of surfactants, i.e. neutral,

*Table 1. Effect of diverse ions on the determination of 300 μg $l^{-1}$ iron(III).*

| Ion added | Tolerance limit* mg $l^{-1}$ |
|---|---|
| Nb(V), V(V). $F^-$ | 7 |
| Co(II), Mo(VI), Cu(II), Sb(III), Tl(III), Bi(III), Hg(II) | 10 |
| Cr(III), Sn(IV) | 12 |
| Ti(IV), Zn(II) | 15 |
| Al(III), Pb(II), Oxalate | 40 |
| Ni(II), $PO_4^{3-}$ | 50 |
| As(V) | 100 |
| Ca(II), Mg(II) | 200 |
| $NO_3^-$, tartrate | 600 |

* Causing error $\leqslant$ 2%.

*Table 2. Analysis of iron in rain water.*

| | | Concentration of Fe in rain water found by | | | | | |
|---|---|---|---|---|---|---|---|
| | | Present method | | Ferrozine method | | GF-AAS | |
| Site | Date | μg $l^{-1}$ | RSD ±% | μg $l^{-1}$ | RSD ±% | μg $l^{-1}$ | RSD ±% |
| Raipur | | | | | | | |
| | 05-01-95 | 89·6 | 1·2 | 90·2 | 1·4 | 91·4 | 4·4 |
| | 11-07-95 | 44·8 | 1·1 | 45·0 | 1·2 | 42·6 | 5·0 |
| Korba | | | | | | | |
| | 04-04-95 | 53·2 | 1·0 | 53·0 | 1·3 | 52·9 | 4·5 |
| | 22-07-95 | 36·4 | 1·3 | 37·0 | 1·2 | 36·6 | 4·6 |
| Bilaspur | | | | | | | |
| | 26-03-95 | 67·2 | 1·1 | 67·5 | 1·0 | 67·0 | 5·0 |
| | 10-07-95 | 53·2 | 1·1 | 53·4 | 1·1 | 53·1 | 5·0 |
| Bhilai | | | | | | | |
| | 17-02-95 | 86·8 | 0·9 | 88·0 | 1·1 | 87·2 | 4·8 |
| | 08-07-95 | 42·0 | 1·1 | 42·0 | 1·0 | 42·4 | 3·6 |
| Bhatapara | | | | | | | |
| | 26-02-95 | 67·2 | 0·8 | 66·9 | 1·0 | 67·0 | 3·5 |
| | 24-07-95 | 140·0 | 1·0 | 139·4 | 1·1 | 139·7 | 5·0 |
| Ambikapur | | | | | | | |
| | 20-06-95 | 89·6 | 1·2 | 90·3 | 1·5 | 90·0 | 3·9 |
| | 30-07-95 | 44·8 | 1·2 | 45·0 | 1·1 | 45·0 | 4·0 |
| Pithora | | | | | | | |
| | 30-05-95 | 78·4 | 1·1 | 80·0 | 1·0 | 79·4 | 5·0 |
| | 02-08-95 | 39·2 | 1·0 | 39·2 | 1·2 | 39·8 | 5·6 |
| Gariyaband | | | | | | | |
| | 11-03-95 | 67·2 | 0·7 | 67·5 | 0·9 | 67·1 | 4·9 |
| | 11-07-95 | 44·8 | 0·9 | 44·6 | 1·0 | 45·0 | 5·0 |
| Kanker | | | | | | | |
| | 06-05-95 | 67·2 | 1·0 | 67·3 | 1·1 | 67·2 | 3·8 |
| | 12-07-95 | 61·6 | 1·1 | 62·0 | 1·6 | 61·8 | 4·0 |
| Raigarh | | | | | | | |
| | 15-05-95 | 78·4 | 1·3 | 78·5 | 1·1 | 78·6 | 5·0 |
| | 17-07-95 | 50·4 | 1·0 | 50·1 | 1·4 | 51·0 | 4·5 |
| Dallirajhara | | | | | | | |
| | 26-02-95 | 72·8 | 1·4 | 72·0 | 1·2 | 72·6 | 4·6 |
| | 09-07-95 | 56·0 | 1·1 | 55·9 | 1·0 | 56·3 | 4·0 |
| Chirmiri | | | | | | | |
| | 20-06-95 | 50·6 | 1·0 | 50·2 | 1·1 | 51·0 | 4·3 |
| | 05-08-95 | 28·0 | 0·5 | 27·8 | 0·9 | 28·1 | 3·6 |
| Janjgir | | | | | | | |
| | 10-05-95 | 67·2 | 1·0 | 67·1 | 1·1 | 67·2 | 5·1 |
| | 01-08-95 | 44·8 | 0·9 | 44·9 | 1·0 | 45·2 | 4·8 |

cationic, and anionic, on the peak height of signal were also tested (figure 1). The signal peak height was found to increase as the basicity of the surfactant increased. Peak height increases slightly with neutral and anionic surfactants, i.e. Triton X-100 (TX-100), Triton X-300 (TX-300), sodium lauryl sulphate (SLS), lithium lauryl sulphate (LLS) and BRIJ 30 (polyoxyethylene(4) lauryl ether). The highest signal peak height was recorded with cationic surfactants (CTAB and CPC), but with CTAB no smooth base line was traced out due to the formation of a least soluble yellowish species, either with thiocyanate ions or the complex. At least $1{\cdot}6 \times 10^{-3}$ M CPC was needed to get the maximum peak height and its further addition up to $3{\cdot}0 \times 10^{-3}$ M had no adverse effect either on the signal peak height or the peak resolution. There was a noticeable synergism in terms of hyperchromic shift of the complex in the presence of CPC; this was found to be 5·0 in the presence of CPC (figure 1).

*Sensitivity, optimum concentration, and statistics*

The value of apparent molar absorptivity of the complex in terms of iron is $(2{\cdot}00) \times 10^{4}$ l mole$^{-1}$ cm$^{-1}$ at the absorption maximum of 490 nm. The detection limit (amount causing more absorbance than three times standard deviation) of the method is 8·0 ppb iron. A calibration curve was prepared by plotting either absorbance or peak height versus concentration of iron in ppb injected followed linearity over 30–3000 ppb iron. The slope, intercept, and correlation coefficient of the calibration curve recorded (at gain factor = 5 over the range of 30–800 ppb iron) were found to be $1{\cdot}90 \times 10^{-2}$,

*Table 3. Iron flux precipitated during hydrological year,* 1995 *in central India.*

| Site | Iron flux, μg/m²/s (No. of events) | | | | | | | | |
|---|---|---|---|---|---|---|---|---|---|
| | January | February | March | April | May | June | July | August | September |
| Raipur | 0·14 (1) | 0·02 (1) | 0·46 (4) | 0·21 (2) | 0·89 (6) | 2·77 (8) | 1·84 (14) | 1·26 (12) | 0·84 (6) |
| Korba | — | — | 0·18 (2) | 0·01 (1) | 0·30 (1) | 0·23 (1) | 0·41 (5) | 0·70 (3) | 0·60 (3) |
| Bhilai | — | 0·04 (1) | 0·18 (5) | 0·52 (3) | 0·71 (5) | 0·17 (2) | 0·61 (15) | 0·26 (4) | — |
| Bilaspur | — | 0·09 (1) | 0·05 (2) | 0·13 (1) | 0·67 (4) | 1·29 (4) | 1·48 (12) | 0·68 (13) | 0·20 (1) |
| Gariyaband | — | 0·08 (1) | 0·16 (3) | 0·48 (4) | 0·64 (3) | 0·29 (4) | 0·58 (10) | 0·45 (3) | — |
| Kanker | — | 0·07 (1) | — | — | 0·31 (3) | 0·71 (1) | 0·91 (8) | 0·63 (6) | 0·20 (2) |
| Dallirajhara | — | 0·05 (1) | 0·12 (2) | — | 0·16 (1) | 0·40 (2) | 0·55 (7) | 0·25 (5) | — |
| Janjgir | — | — | 0·01 (1) | — | 0·06 (1) | 0·23 (2) | 0·49 (3) | 0·27 (6) | 0·07 (2) |
| Raigarh | — | — | — | — | 0·23 (2) | — | 0·23 (4) | 0·58 (5) | 0·28 (4) |
| Chirmiri | — | — | — | — | — | 0·15 (1) | 0·15 (4) | 0·23 (6) | 0·11 (2) |
| Pithora | — | — | — | — | 0·16 (2) | 0·10 (1) | 0·30 (3) | 0·07 (2) | — |
| Bhatapara | — | 0·05 (1) | 0·12 (2) | — | — | 0·05 (1) | 0·80 (4) | 0·22 (1) | — |
| Ambikapur | — | — | — | — | — | 0·29 (1) | 0·78 (6) | 0·31 (4) | 0·17 (1) |

*Table 4. Speciation of iron in the rain water of central India during hydrological year,* 1995.

| Site | Ratio $Fe^{2+}/Fe^{3+}$ | | | | | | | | |
|---|---|---|---|---|---|---|---|---|---|
| | January | February | March | April | May | June | July | August | September |
| Raipur | 7·0 | 3·6 | 2·0 | 4·4 | 8·3 | VH | VH | VH | VH |
| Korba | — | — | 5·7 | 2·2 | 8·3 | VH | VH | VH | VH |
| Bilaspur | — | 3·0 | 3·9 | 3·4 | 4·9 | VH | VH | VH | VH |
| Bhilai | — | 2·8 | 2·9 | 4·6 | 2·4 | VH | VH | VH | VH |
| Bhatapara | — | 5·0 | 3·6 | — | — | VH | VH | VH | VH |
| Ambikapur | — | — | — | — | — | VH | VH | VH | VH |
| Pithora | — | — | — | — | 3·6 | VH | VH | VH | VH |
| Gariyaband | — | 3·0 | 2·5 | 2·7 | 6·0 | VH | VH | VH | VH |
| Kanker | — | 1·3 | — | — | 3·7 | VH | VH | VH | VH |
| Raigarh | — | — | — | — | 7·0 | VH | VH | VH | VH |
| Dallirajhara | — | 3·3 | 3·0 | — | 21·5 | VH | VH | VH | VH |
| Chirmiri | — | — | — | — | — | VH | VH | VH | VH |
| Janjgir | — | — | 10·0 | — | 5·0 | VH | VH | VH | VH |

VH = very high.

$-8{\cdot}90 \times 10^{-2}$, and $+0{\cdot}999$, respectively. The RSD of six different samples for 300 ppb Fe (at gain factor = 5) was ±0·9%.

*Effect of diverse ions.* The effect of diverse ions on the determination of iron was examined by separately injecting solutions containing 300 ppb $Fe^{3+}$. No interference in the determination of iron was discovered: tolerance limits are shown in table 1.

*Comparison of the method.* Flame AAS was tested for the detection of iron in rain water but the sensitivity was very poor. Therefore the graphite furnace AAS and ferrozine methods were used to check the validity of the present method (see table 2). RSD with the graphite furnace AAS was found to be higher over a narrow optimum concentration range. In addition, iron could not be quantified with it. The ferrozine reagent is more

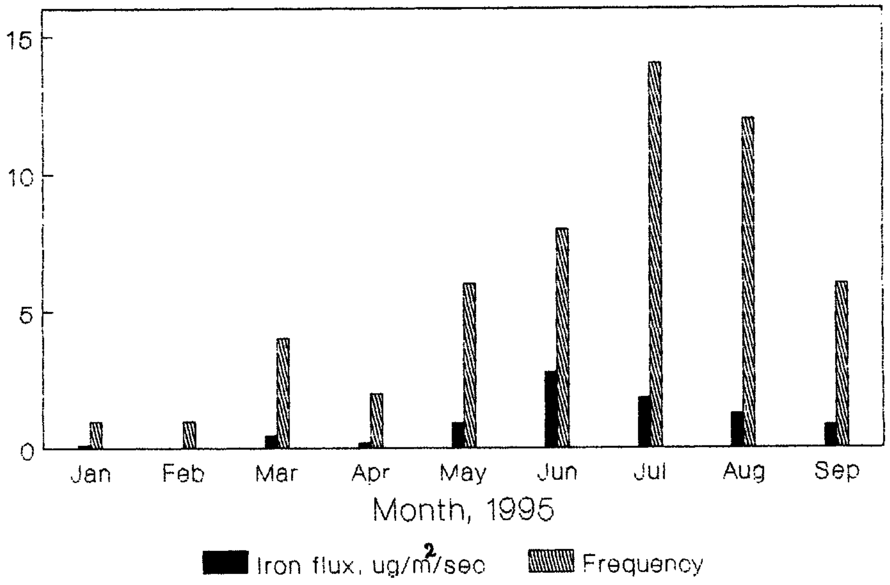

*Figure 2. Seasonal variation in iron flux precipitated in Raipur.*

expensive than thiocyanate and the signal peak height was affected by some analytical variables (acidity variations and matrix interferences). The data obtained by the three methods were found to be comparable (table 2).

*Precipitation and speciation of iron*

The method described was used to characterize soluble iron precipitated in central India during hydrological year 1995. The present method was used directly to detect ferric iron in rain water; total iron was measured after prior oxidation with a few drops of nitric acid. Ferrous iron was computed by subtracting the concentration of ferric iron from the total iron. A remarkable seasonal and spatial variation in the iron flux precipitated was observed (see table 3). The average flux precipitated at 13 sampling sites was in the range of 0·16–0·94 μg $Fe/m^2/s$. The precipitation of iron was found to be highest during May to July. After July, the iron flux precipitated decreases gradually (see figure 2). Between May and July, it is likely that iron is from natural sources because wind speeds are high and a lot of dust is blown around. The redox chemistry of iron in India's tropical climate was found to be very different to temperate climates [4]. Between January and May, the ratio of Fe(II)/Fe(III) among 13 sampling stations was in the range of 1·3–21·5, whereas between June and September almost all soluble iron was in the reduced form (see table 4). The sources of iron differ from region to region; in industrial belt between Bhilai and Raigarh iron in the rain water probably comes largely from anthropogenic sources (steel plants, cement plants, coal ash), whereas in the south (Kanker, Pithora and Gariyaband) iron comes mostly from natural sources because the soil is heavily loaded with iron minerals. Similarly, in Korba and Chirmiri, iron is expected to come mainly from the fly ash emitted by thermal power plants, whereas in north (Ambikapur) for example it is mostly from distant sources because the area is situated in the path of the monsoon.

### Conclusion

The present method has been shown to be simple, rapid, selective and sensitive. It compares well with most of the methods reported for the monitoring of iron in water samples. It could be used as a laboratory method for the trace analysis of iron in a variety of materials, for example environmental, clinical, pharmaceutical, biological and agricultural.

### Acknowledgements

The authors wish to thank DST, New Delhi, India and the Alexander-von Humboldt Foundation, Bonn, Germany for financial support.

### References

1. Morales C., *Saharan Dust–Mobilization, Transport, Deposition* (John Wiley, New York, 1979).
2. Hansen, L. D., Silberman, D., Fisher, G. L. and Eatough, D. J., *Environ. Sci. Technol.*, **18** (1984), 181.
3. Weschler, C. J., Mandich, M. L. and Graedel, T. E., *Journal of Geophysical Research*, **91** (1986), 5189.
4. Pandis, S. N. and Seinfeld, J. H., *Journal of Geophysical Research*, **94** (1989), 12911.
5. Pandis, S. N., Seinfeld, J. H. and Pilinis, C., *Atmos. Environ.*, **26A** (1992), 2509.
6. Berg, T., Royset, O. and Steinnes, E., *Atmos. Environ.*, **28** (1994), 3519.
7. Marin, S. R., Olave, S. G., Andonie, O. E. and Arlegul, O. G., *International Journal of Environmental Analytical Chemistry*, **52** (1993), 127.
8. Slavin, W., Manning, D. C. and Caenrick, G. R., *Atomic Spectroscopy*, **2** (1981), 137.
9. Hansson, H., Ekholm, A. P. and Ross, H. B., *Environmental Science Technology*, **22** (1988), 527.

10. Bozsal, G., Schlemmer, G. and Grobenski, Z., *Talanta*, **37** (1990), 545.
11. Tecator, A. B., *Introduction to the Use of Flow injection Analysis (FIA)* (Sweden, 1988).
12. Wade, H., Nakagawa, G. and Ohshita, K., *Analytica Chimica Acta*, **153** (1983), 199.
13. Ishi, H., Aoki, M., Aita, T. and Odashima, T., *Analytical Science*, **2** (1986), 125.
14. Yammane, T. and Yamnla, H., *Analytica Chimica Acta*, **308** (1995), 433.
15. Blain, S. and Treguer, P., *Analytica Chimica Acta*, **308** (1995), 425.
16. Krekler, S., Frenzel, W. and Schulz, G., *Analytica Chimica Acta*, **296** (1994), 115.
17. Kolotyrkina, I. Y., Shpitun, L. K., Zolotav, Y. A., and Malahoff, A., *Analyst*, **120** (1995), 201.

